# New Mode of Producing Oxygen

**Published:** 1869-09

**Authors:** 


New Mode of Producing Oxygen.—Messrs. Montmagnon and Delaire produce oxygen from the atmosphere by means of charcoal and water, or by saline solutions. They state that 100 litres of fresh charcoal, when exposed to atmospheric air, will absorb 925 litres of oxygen and only 705 litres nitrogen. If the charcoal so saturated with gas is then saturated with water there will be expelled 650 litres of nitrogen and only 350 litres of oxygen. Thus 575 litres of oxygen and only 45 litres of nitrogen are left in the charcoal. These gases they remove by means of an air pump, when the charcoal is again ready to absorb oxygen and nitrogen from the air. The oxygen thus obtained is pure enough for all ordinary purposes, but the cost of procuring it by this method has not yet been practically determined.

